# Retraction Note: Enhancement of oxidation resistance via a self-healing boron carbide coating on diamond particles

**DOI:** 10.1038/s41598-022-26781-z

**Published:** 2022-12-22

**Authors:** Youhong Sun, Qingnan Meng, Ming Qian, Baochang Liu, Ke Gao, Yinlong Ma, Mao Wen, Weitao Zheng

**Affiliations:** 1 College of Construction Engineering, Jilin University, Changchun, 130026 People’s Republic of China; 2 Key Lab of Drilling and Exploitation Technology in Complex Conditions, Ministry of Land and Resources, Changchun, 130061 People’s Republic of China; 3 State Key Laboratory of Superhard Materials, Jilin University, Changchun, 130012 People’s Republic of China; 4 Department of Materials Science, Jilin University, Changchun, 130012 People’s Republic of China

Retraction of: *Scientific Reports* 10.1038/srep20198, published online 02 February 2016

Editors have retracted this Article. Concerns were raised regarding the data and figures in this article. Specifically some of the results originating from different samples have the same noise background, e.g. in Figure 1 but also in other figures. These type of similarities are unlikely to occur by chance for the type of experiments reported here. Additionally, some of this data has been published later in^1^ by overlapping authors where it is described as representing different samples. Specifically, data for sample D3 in Figure 1 in this Article is the same as data in Figure 2 in^1^; data in Figure 2a in this Article appears identical to data in Figure 4a in^1^ with the exception that data in this Article contains additional C-C peaks that seem to have been removed in^1^; data in Figure 7a in this Article appears to be identical to data in Figure 4b in^1^ with the exception of being shifted by approximately 6eV. The Authors are not able to provide the original data due to the time that has passed since publication. The Editors no longer have confidence in the data reported in this Article.

Qingnan Meng agrees with the retraction and its wording. The Editors were not able to obtain current contact details for Youhong Sun, Ming Qian, Baochang Liu, Ke Gao, Yinlong Ma, Mao Wen & Weitao Zheng.
